# Acceptability of Guidelines to Stop Colon Cancer Screening by Estimated Life Expectancy

**DOI:** 10.1001/jamanetworkopen.2024.47802

**Published:** 2024-12-03

**Authors:** Laura E. Brotzman, Brian J. Zikmund-Fisher, Eve A. Kerr, Mohammed Kabeto, Jeffrey T. Kullgren

**Affiliations:** 1 Department of Health Behavior and Health Equity, University of Michigan School of Public Health, Ann Arbor; 2 Department of Internal Medicine, University of Michigan, Ann Arbor; 3 Veterans Affairs (VA) Center for Clinical Management Research, VA Ann Arbor Healthcare System, Ann Arbor, Michigan

## Abstract

This cross-sectional study examines whether older adults with limited vs longer estimated life expectancy accept guideline recommendations that patients stop getting screened for colon cancer once they reach age 75 years.

## Introduction

Recent professional guidelines have sought to deimplement use of care that generally does not improve patient outcomes and involves unnecessary harms or risks (ie, low-value care). One example is guidelines that recommend patients older than 75 years stop getting screened for colon cancer.^1^ The benefit-cost tradeoff for such recommendations varies across patients. For example, older adults with more health problems and limited life expectancy receive less benefit from cancer screening than healthier patients of the same age. We examined whether older adults with limited vs longer estimated life expectancy accepted such deimplementation guidelines.

## Methods

In this cross-sectional study, we used data from Module 8 of the 2018 wave of the Health and Retirement Study (HRS),^2^ an ongoing biennial longitudinal cohort study of approximately 20 000 US adults 50 years and older conducted by the University of Michigan, Ann Arbor. HRS data are weighted to reflect the US population of older adults; module recipients are a random sample of each HRS wave. According to University of Michigan policy, this work does not qualify as human participants research and is not subject to ethics review. This report follows STROBE reporting guidelines.

Data were analyzed from January 25, 2023, to September 10, 2024. We used Stata, version 18.5.5 (Stata Corp, LLC), to analyze responses to the following: “Guidelines recommend that patients stop getting screened for colon cancer once they reach age 75. This is because for many healthy patients age 75 and older, the harms of testing may be greater than the benefits of finding a new cancer. How acceptable is this recommendation to you personally?” (Response options include very unacceptable, somewhat unacceptable, somewhat acceptable, or very acceptable). The threshold for statistical significance was 2-tailed *P* < .05.

## Results

The sample included 1302 respondents. After excluding 28 respondents missing 1 or more items, the total analytic sample included 1273. We conducted a subgroup analysis among 304 respondents (23.9%) with limited estimated life expectancy, defined as a score of 8 or more on the Lee Index for 10-year mortality, and 969 (76.1%) with longer life expectancy, defined as a score of 7 or lower on the Lee Index.^3^ Most respondents were female (794 [61.0%] vs 508 [39%] male), 407 (31.3%) were aged 50 to 59 years, and 403 (31.0%) were aged 60 to 69 years (Table).

**Table.** Estimated Population Characteristics

Characteristic	No. of respondents	Weighted % (95% CI)
Age (n = 1302)		
50-59	407	31.3 (28.8-33.8)
60-69	403	31.0 (28.5-33.5)
70-79	278	21.4 (19.2-23.7)
≥80	214	16.4 (14.5-18.6)
Sex (n = 1302)		
Female	794	61.0 (58.3-63.6)
Male	508	39.0 (36.4-41.7)
Lee Index (n = 1273)^a^		
0	82	6.4 (5.2-7.9)
1	83	6.5 (5.3-8.0)
2	149	11.7 (10.0-13.6)
3	139	10.9 (9.3-12.8)
4	144	11.3 (9.7-13.2)
5	151	11.9 (10.2-13.8)
6	122	9.6 (8.1-11.3)
7	99	7.8 (6.4-9.4)
8	90	7.1 (5.8-8.6)
9	59	4.6 (3.6-5.9)
10	48	3.8 (2.9-5.0)
11	42	3.3 (2.5-4.4)
12	23	1.8 (1.2-2.7)
13	16	1.3 (0.8-2.0)
14	13	1.0 (0.6-1.8)
≥15	13	1.0 (0.6-1.8)

^a^ The Lee index includes the following factors: age, sex, body mass index, history of diabetes, cancer, lung disease, heart failure, smoking, difficulty bathing, difficulty managing finances, difficulty walking, and difficulty pushing or pulling. Because this index includes many factors associated with life expectancy, (ie, potential confounding variables that would be difficult to control for), only basic descriptive analyses are reported. An index of 8 or greater indicates less than 10 years of estimated life expectancy.

While a weighted estimate of 60.4% of respondents rated guidelines on stopping colon cancer screening at age 75 years as acceptable, 39.6% found such guidelines somewhat or very unacceptable (Figure). Unacceptable responses were almost identical across the life expectancy subgroups (limited: 39.2%; longer: 39.7%). In exploratory logistic regression analyses, acceptability was lower among male respondents with limited life expectancy but the pattern reversed among female respondents (Lee Index for female sex odds ratio, 1.89 [95% CI, 1.09-3.26]).

**Figure.**
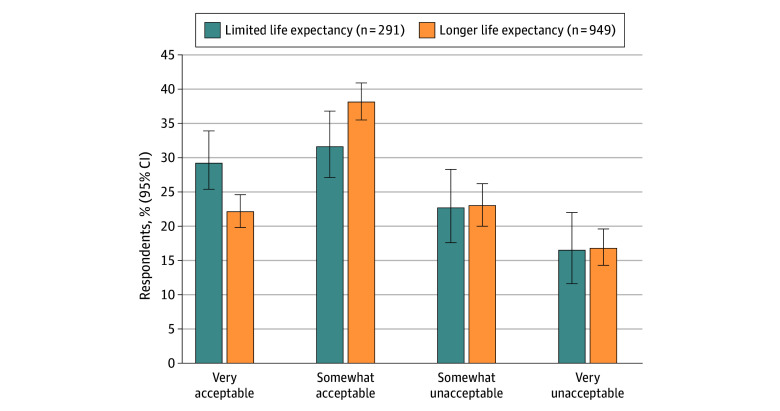
Comparison of Acceptability of Guidelines Recommending Against Colon Cancer Screening After 75 Years of Age Longer life expectancy indicates 10 years or longer; limited life expectancy, less than 10 years.

## Discussion

While many older adults found guidelines limiting colon cancer screening after 75 years of age to be somewhat or very acceptable, a sizable minority did not, and this finding is consistent across respondents with different life expectancies. Limitations of our analysis include reliance on self-report health status and a limited sample size. Nevertheless, these data suggest that limited life expectancy (and the health issues associated with that metric) does not increase older adults’ acceptance of guidelines that seek to deimplement low-value care, although less healthy individuals are most likely to benefit and/or avoid unnecessary harms by following such guidelines.

## Notes

Supplement.
Data Sharing Statement
