# Supplementary material for: Acceptability of Guidelines to Stop Colon Cancer Screening by Estimated Life Expectancy
Source: JAMA Netw Open. 2024 Dec 3;7(12):e2447802. doi: 10.1001/jamanetworkopen.2024.47802 (PMC11615702; doi:10.1001/jamanetworkopen.2024.47802)
Supplement: Supplement. — Data Sharing Statement [file jamanetwopen-e2447802-s001.pdf]

Data Sharing Statement

Brotzman. Acceptability of Guidelines to Stop Colon Cancer Screening by Estimated Life Expectancy. *JAMA Netw Open*. Published December 03, 2024.
doi:10.1001/jamanetworkopen.2024.47802

Data
Data available: Yes
Data types: Deidentified participant data, Data dictionary
How to access data: https://hrs.isr.umich.edu/
When available: With publication

Supporting Documents
Document types: None

Additional Information
Who can access the data: Public data is available to anyone who registers for an HRS account.
Types of analyses: For any purpose.
Mechanisms of data availability: Public data is available to anyone who registers for an HRS account, sensitive health data files available are from the public data portal after a supplemental agreement is signed, and HRS restricted data files require a detailed application process, and are available only through remote virtual desktop or encrypted physical media.
